# MicroRNAs in Diabetic Nephropathy: From Biomarkers to Therapy

**DOI:** 10.1007/s11892-016-0724-8

**Published:** 2016-03-14

**Authors:** Kate Simpson, Alexa Wonnacott, Donald J. Fraser, Timothy Bowen

**Affiliations:** Wales Kidney Research Unit, School of Medicine, College of Biomedical and Life Sciences, Cardiff University, Heath Park, Cardiff, CF14 4XN UK

**Keywords:** Diabetic nephropathy, MicroRNA, Biomarker, Therapeutics, Fibrosis, Non-coding RNA

## Abstract

Recent estimates suggest that 1 in 12 of the global population suffers from diabetes mellitus. Approximately 40 % of those affected will go on to develop diabetes-related chronic kidney disease or diabetic nephropathy (DN). DN is a major cause of disability and premature death. Existing tests for prognostic purposes are limited and can be invasive, and interventions to delay progression are challenging. MicroRNAs (miRNAs) are a recently described class of molecular regulators found ubiquitously in human tissues and bodily fluids, where they are highly stable. Alterations in miRNA expression profiles have been observed in numerous diseases. Blood and tissue miRNAs are already established cancer biomarkers, and cardiovascular, metabolic and immune disease miRNA biomarkers are under development. Urinary miRNAs represent a potential novel source of non-invasive biomarkers for kidney diseases, including DN. In addition, recent data suggest that miRNAs may have therapeutic applications. Here, we review the utility of miRNAs as biomarkers for the early detection and progression of DN, assess emerging data on miRNAs implicated in DN pathology and discuss how the data from both fields may contribute to the development of novel therapeutic agents.

## Introduction

Diabetic nephropathy (DN) is a complication of type 1 (T1DM) and type 2 diabetes mellitus (T2DM), the incidence of which is increasing, with an estimated 9 % of the adult population affected globally [1]. DN develops in approximately 40 % of patients with T1DM. DN is also common in those with T2DM, who are also at risk of renal dysfunction from other pathologies including ischaemic nephropathy. DN is the leading cause of renal failure requiring renal replacement therapy worldwide [2], but effective methods to identify and halt progression of pathophysiological changes of DN remain elusive.

Numerous risk factors for the development of DN have been postulated, including ethnicity and inherited genetic differences [3, 4]. Downstream contributors include hyperglycaemia and insulin resistance, and aberrant haemodynamics leading to intraglomerular hypertension and hyperfiltration.

Hyperglycaemia is associated with the generation of advanced glycation end products, renin-angiotensin system activation, increased cytokine production (most notably pro-fibrotic transforming growth factor (TGF)-β), reactive oxygen species and protein kinase C activity [5, 6]. Insulin resistance is associated with loss of endothelial and vascular modulation via nitric oxide and NF-κB beta pathways, with defects in podocyte-specific insulin signalling recently implicated in early DN [7]. The histological consequences of such insults include mesangial hyperexpansion, nodular glomerulosclerosis and tubulointerstitial fibrosis [8], the degree of which correlates to loss of glomerular filtration rate and, ultimately, renal failure [9].

## MicroRNAs

MicroRNAs (MiRNAs) are ubiquitous endogenous, non-coding, single-stranded (ss)RNA transcripts, most frequently of 19–25 nucleotides in length, that act as post-transcriptional regulators of gene expression by blocking protein translation and/or inducing messenger RNA (mRNA) degradation. It is currently estimated that miRNAs regulate the expression of at least 60 % of all protein coding genes, and alterations in miRNA expression profiles have been observed in numerous pathological processes. Consequently, there is much current interest in miRNAs both as novel biomarkers and as potential targets for therapeutic intervention.

The first miRNA, lin-4, was identified in 1993 in the nematode *Caenorhabditis elegans* [10], the significance of this finding becoming clearer when a second miRNA, let-7, was also detected [11], and lin-4 and let-7 were found to be highly conserved in eukaryotes, suggesting an important functional role [12, 13]. Over 1000 human miRNAs have now been identified through bioinformatic and molecular cloning approaches, although functional validation has not yet been established in every case.

### Biogenesis and Functional Mechanism of MicroRNA-Mediated Translational Repression

**Fig. 1:**
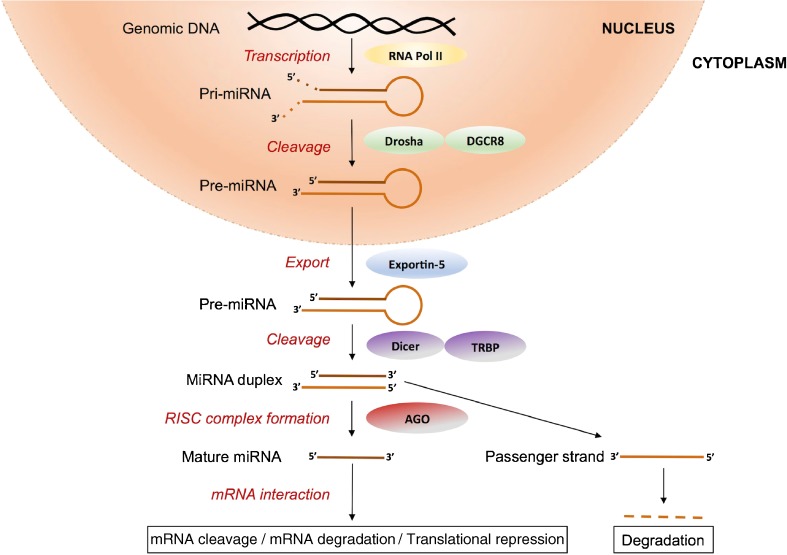
MicroRNA biogenesis and repression of gene expression

As shown in Fig. 1, canonical miRNA biogenesis begins with nuclear transcription, most frequently by RNA polymerase II, into transcripts known as primary miRNAs (pri-miRNAs). Pri-miRNAs vary in length, in some cases spanning kilobases, and have a distinctive stem-loop structure. They are cleaved within the nucleus into precursor miRNAs (pre-miRNAs) by a multiprotein complex that includes the ribonuclease III (RNase III) enzyme drosha and the cofactor Di George syndrome critical region gene 8 (DGCR8). DGCR8 binds to pri-miRNAs at a specific distance from the ssRNA-double-stranded (dsRNA) junction at the base of the stem-loop [14] and directs drosha to cleave the pri-miRNA 11 nucleotides from the ssRNA-dsRNA junction to form a 70–100 bp pre-miRNA [15]. Pre-miRNAs have a distinctive hairpin structure that is recognised by exportin-5, a dsRNA-binding protein that facilitates egression of pre-miRNAs from nucleus to cytoplasm. Subsequent cytoplasmic modification involves pre-miRNA cleavage to form mature ds miRNAs in a miRNA/miRNA* duplex consisting of two ss miRNAs referred to as the guide strand (miRNA) and the passenger strand (miRNA*). Pre-miRNA cleavage is facilitated by dicer, another RNase III enzyme within a large multiprotein complex that, in this case, contains the TAR RNA-binding protein (TRBP), which binds dsRNA and guides dicer to the correct cleavage site.

The function of miRNAs in silencing gene expression follows incorporation into the RNA-induced silencing complex (RISC), which includes the argonaute (AGO) proteins, after which the miRNA* is typically degraded. The RISC-bound miRNA then associates with the 3′-untranslated region (3′-UTR) of its target mRNA via classical Watson-Crick base pairing. Perfect sequence complementarity between miRNA and target mRNA 3′-UTR results in mRNA cleavage by AGO2, and imperfect complementarity results in translational repression and/or degradation of the mRNA target [16].

## Utility of MicroRNAs as Diabetic Nephropathy Biomarkers

### Existing Diabetic Nephropathy Biomarkers

Current DN diagnosis and monitoring of disease progression rely heavily on the detection of urinary microalbuminuria. However, not all patients with microalbuminuria progress to overt proteinuria and nephropathy, complicating prognosis. In addition, tissue damage and induction of inflammation have already occurred by the time that microalbuminuria is detectable. Furthermore, microalbuminuria is not specific to DN but is merely a hallmark of glomerular, and more specifically podocyte, dysfunction. Biopsy is the present diagnostic and prognostic test for intrinsic renal disease, but this highly invasive and expensive procedure has a 3 % risk of major complications.

There is therefore powerful incentive for research teams and industrial collaborators to develop improved prognostic and diagnostic tests for DN based on new biomarkers that provide earlier warning, more specific information on disease progression and, ideally, response to treatment. Pragmatism must be exercised in any biomarker screening programme, with downstream applicability borne in mind throughout. Consideration of test cost, speed and feasibility of entry into existing treatment pathways is essential to optimise use of resources. Nevertheless, the identification of differences in miRNA expression that do not have utility for biomarker analyses may still provide important information on disease mechanism, DN pathology and the identification of potential targets for therapy. The latter areas will be discussed later in this review.

### Disease-Specific MicroRNA Signatures and Tissue Specificity

Expression profiling of miRNAs was first carried out in studies on carcinogenesis. Calin and colleagues showed that specific miRNAs were downregulated in circulating mononuclear cells from chronic lymphocytic leukaemia patients and identified a 13-miRNA expression profile associated with prognostic factors and disease progression [17, 18]. Tissue-specific miRNA expression has been reported, with perhaps the best-known example being the discovery that miR-122 expression issue was restricted to the mouse liver [19]. Mapping of miRNA expression in human tissues has since been reported, including profiles from cells in the different nephron regions [20].

### Circulating MicroRNAs

Detection of circulating miRNAs offers a quicker, potentially automatable alternative method for DN diagnosis to biopsy and tissue analysis. Extracellular miRNAs were first reported in cell culture medium in an in vitro study that identified exosomal miRNAs that were transferred between mast cells, suggesting that cell-cell communication could be facilitated by fluid-borne miRNAs [21].

Significant differences in biological fluid miRNA expression profiles have now been observed in numerous disease states. In 2008, miRNAs were first detected in plasma and serum [22, 23]. Mitchell and colleagues identified circulating stable, readily detectable miRNAs in the blood of healthy human donors and patients with metastatic prostate cancer [22]. Detection of miR-141 in serum differentiated these two groups, underlining the potential of miRNAs for use as high-throughput diagnostic biomarkers [22]. Similarly, Lawrie and co-workers demonstrated the diagnostic potential of a miRNA panel composed of miR-21, miR-155 and miR-210 in diffuse large B cell lymphoma patients [23]. As reviewed by Allegra and colleagues, blood miRNAs are already established cancer biomarkers, and cardiovascular, metabolic and immune disease miRNA biomarkers are under development [24].

Stability is a key consideration when evaluating the use of any potential biomarker. Characterisation of blood-borne miRNAs by centrifugation and size exclusion chromatography identified two populations of circulating miRNAs: extracellular vesicle-associated (EVA-) and non-vesicle-associated (NVA-) miRNAs [25•], and much work has since been devoted to identifying the factors stabilising fluid-borne miRNAs, as well as the functions of these transcripts.

In human plasma, association of extracellular miRNAs with AGO proteins results in the formation of ribonucleoprotein complexes with the RISC that stabilise these transcripts (Fig. 1), and specific association of selected miRNAs with AGO2 has been demonstrated in plasma [26]. This association protects miRNAs from degradation in RNase-rich biological fluids such as blood [25•, 26, 27].

The role of extracellular vesicles as miRNA transporters that mediate signalling has been proposed [28]. Studies by Wang et al. correlated an increase in detectable NVA-miRNAs during serum-deprived cellular stress, and suggested that their secretion might drive cell regulatory mechanisms via miRNA-regulated cell-cell signalling [29]. The role of high-density lipoproteins in transporting functional NVA-miRNAs has also been posited [30].

### Urinary MicroRNAs

The use of urinary miRNAs as disease biomarkers provides the additional advantages of non-invasive testing for which samples can be collected remotely and mailed to the test laboratory, as well as the possibility of a rapid point-of-care test. Recent in-depth studies have analysed the stability of urinary miRNAs [31–33]. Extremes of pH, prolonged storage at room temperature, multiple freeze-thaw cycles and subjection to RNase activity have all been used in these assessments, and we have recently demonstrated that urinary miRNAs are stabilised by association with AGO2 and exosomes [33]. Robust techniques for detection of miRNAs in urine and in urinary sediment have now been established [31–33], but more rapid and higher-throughput protocols are likely to be required for routine clinical testing.

Differential urinary miRNA expression profiles have been reported in kidney disease. One study showed decreased detection of miR-21 and miR-29, and increased miR-93 detection, in urine samples from IgA nephropathy patients compared to controls [32]. Argyropoulos et al. identified a panel of 27 differentially regulated urinary miRNAs that varied with DN progression [34].

The definition of the relative contributions of EVA- and NVA-miRNA populations to the total miRNA complement of each body fluid, the miRNA composition of these populations, and the functionality of miRNAs in each population is presently the subject of much ongoing research. It has been reported that serum and salivary miRNAs reside primarily in exosomes [35]. However, studies on plasma [25•, 26] as well as seminal fluid, dendritic cells, mast cells and ovarian cancer cells [36] contend that the majority of extracellular miRNAs are NVA. Furthermore, it has been suggested that the numbers of EVA-miRNAs are insufficient to mediate signalling [36]. Our recent work has provided definitive evidence of association of EVA urinary miR-16 and miR-192 with exosomes and NVA miR-16 and miR-192 with AGO2 [33].

## MicroRNAs in Fibrosis and Diabetic Nephropathy Pathogenesis

### MiR-192 and Fibrosis

As mentioned above, TGF-β is a key cytokine with fundamental importance in DN due to its roles in fibrosis and scarring. TGF-β signalling is regulated by miR-21, which can in turn regulate mature miR-21 expression [37], while our analysis of the TGF-β1 3′-UTR has shown evidence of post-transcriptional regulation by miR-744 [38].

An early step in fibrogenesis in DN involves the repression of E-cadherin by TGF-β1. Kato and colleagues observed that, in the early stages of renal injury, mouse mesangial cells treated with TGF-β1 showed upregulated expression of miR-192 and collagen alpha-2(I) [39]. Conversely, studies from this laboratory found decreased miR-192 expression in advanced-stage human DN renal biopsy samples accompanied by low estimated glomerular filtration rate and tubulointerstitial fibrosis [40]. In cultured human proximal tubular cells, we showed a similar TGF-β1-driven downregulation of miR-192 expression, while forced expression of this transcript repressed ZEB1 and ZEB2 expression, thereby derepressing E-cadherin and exerting an anti-fibrotic effect [40]. Further studies in diabetic apoE mice by Wang et al. identified a similar pattern of TGF-β1-mediated downregulation of miR-192 leading to E-cadherin inhibition [41]. We have since reviewed the pleiotropic roles of miR-192 in the kidney, with both anti- and pro-fibrotic effects that are apparently cell-type dependent [42].

### MiR-21, miR-200 and miR-29 Families in Diabetic Nephropathy

One of the most abundant miRNAs in human tissues, miR-21, has been studied extensively and implicated in the pathogenesis of a number of malignancies. Similarities of these pathologies with fibrogenesis highlight miR-21 and its mRNA targets as possible candidates for DN progression, and renal miR-21 knockdown suppressed TGF-β1 signalling in a mouse model of T2DM [43•]. Increased miR-21 expression has also been identified in renal transplant patients with fibrotic kidney disease and in the urine of fibrotic patients with IgA nephropathy [32].

Lin and co-workers have recently reported functional interactions of miR-21 and TGF-β signalling by confirming that SMAD7, an inhibitor of TGF-β signalling, is a direct target of miR-21 and targets TGF-β by repressing SMAD7 expression, thus preventing rat renal tubular epithelial cell proliferation in an in vitro DN model [44].

The miR-200 family of miR-200a, miR-200b, miR-200c, miR-429 and miR-141 has been ascribed roles in maintaining epithelial differentiation, suggesting anti-fibrotic functions in DN [45]. Downregulated expression of these transcripts by TGF-β has been shown in cancer cell epithelial-to-mesenchymal transition (EMT) [46]. Conversely, upregulated miR-200b, miR-200c and miR-192 expression was observed in TGF-β-treated mouse mesangial cells [47].

Anti-fibrotic effects in DN have also been reported for miR-29 family members miR-29a, miR-29b and miR-29c. For example, diabetic mice expressing a miR-29a transgene showed improved renal function and better podocyte viability compared to wild-type diabetic mice [48]. Knockdown of miR-29a promoted histone deacetylase activity that lead to podocyte apoptosis, proteinuria and subsequent renal dysfunction [48].

Above, we have summarised studies on expression changes in DN and other renal diseases for the most widely studied miRNAs to date. The identification of miRNAs from biomarker screening studies and from disease model studies both have potential for use as therapeutic targets.

## MicroRNAs as Therapeutic Targets

The majority of therapeutic studies investigating miRNAs have so far focussed on cancer, and miRNAs that regulate oncogene expression are often known as oncomiRs. Therapies targeting repression of single oncogenes using small molecule inhibitors have had limited therapeutic response due to the complexity of carcinogenesis, and this complexity is likely to be mirrored in other multifactorial diseases such as DN.

Targeting oncomiRs has the potential benefit of affecting the expression of multiple mRNAs that are oncomiR targets and therefore may target multiple pathways. For example, miR-34a has multiple target mRNAs that include proto-oncogene c-Met, cell cycle regulator cyclin-dependent kinase 4 and B cell lymphoma 2, an anti-apoptotic oncogenic protein [49]. A miR-34 mimic has been used in mouse models of lung cancer resulting in amelioration of disease progression correlated with a repression of all three targets [50]. Clearly, the possibility of deleterious off-target effects must also be considered.

Recent advances in the treatment of hepatitis C virus (HCV) infection represent the best example to date of a miRNA-based therapy. Chimpanzees treated with a miR-122 antagonist conferred long-lasting repression in HCV viraemia [51].

The need for more successful early-stage treatment options for DN patients remains. As discussed above, many studies are now investigating the potential of miRNAs as DN biomarkers. Identification of these miRNAs and other candidates selected on the basis of function and/or GWAS study outputs will allow investigation of their utility as targets to intervene in disease progression.

Several studies have manipulated miRNA expression in in vivo diabetic models. For example, in streptozotocin-treated diabetic mice, miR-192 was downregulated by a locked nucleic acid-modified anti-miRNA in the renal cortex to improve renal fibrosis symptoms [52, 53]. Similarly, knockdown of miR-29c resulted in the prevention of DN progression in db/db mice [54]. In a rat remnant kidney model of renal fibrosis, low-dose treatment with anti-cancer agent paclitaxel improved renal function, inhibited Smad2/3 activation and downregulated miR-192 expression [55].

Unlike liver-specific expression of miR-122, there are no  renal-specific miRNAs, but ready uptake from the circulation by renal proximal tubular epithelial cells facilitates targeted delivery to the kidney. Ultrasound-microbubble-mediated gene transfer has been used to deliver plasmids to mammalian kidneys [56]. Developed first as ultrasound contrast agents, microbubbles have since been described as ‘theranostic’ agents, with uses in diagnostics and now in therapeutics, depending on the ultrasound parameters used [57]. High-intensity ultrasound causes microbubble oscillation, resulting in ‘inertial cavitation’ and release of the therapeutic agent(s) attached to, or contained within, the microbubbles [57]. Microbubble technology has been used to knockdown miR-21 expression in db/db mice, ameliorating microalbuminuria, renal fibrosis and inflammation [43•].

## Conclusion

Emerging miRNA analyses continue to show promise for these transcripts as both biomarkers and as therapies in renal pathologies [58]. However, considerably more work will be required to consolidate and translate these findings. Biomarker studies of appropriate size and power will be required to augment the studies carried out to date, and animal model studies on mechanism represent the first stage on the long road to therapy. Nevertheless, the identification of miRNAs as novel targets for the improvement of DN patient outcomes provides hope for significant future clinical developments.
